# Contribution of PEPFAR-Supported HIV and TB Molecular Diagnostic Networks to COVID-19 Testing Preparedness in 16 Countries

**DOI:** 10.3201/eid2813.220789

**Published:** 2022-12

**Authors:** Erin Rottinghaus Romano, Katrina Sleeman, Patricia Hall-Eidson, Clement Zeh, Ravikiran Bhairavabhotla, Guoqing Zhang, Amitabh Adhikari, George Alemnji, Yolanda Rebello Cardo, Ana Pinheiro, Barbara Pocongo, Laura T. Eno, Judith D. Shang, Clement B. Ndongmo, Hilda Rosario, Orquidea Moreno, Lucia Aurora De La Cruz De León, Peter Fonjungo, Constantin Kabwe, Steve Ahuke-Mundeke, Dan Gama, Sindisiwe Dlamini, Gugu Maphalala, Tefsay Abreha, Anne Purfield, Yared Tedla Gebrehiwot, Daniel Melese Desalegn, Frank Basiye, Jane Mwangi, Nancy Bowen, Yohannes Mengistu, Shirley Lecher, Elizabeth Kampira, Muluken Kaba, Joseph Bitilinyu-Bangoh, Gillian Masamha, Sofia Omar Viegas, R. Suzanne Beard, Gerhard van Rooyen, Andreas N. Shiningavamwe, McPaul I.J, Nnaemeka C. Iriemenam, Nwando Mba, Catherine Okoi, Joel Katoro, Dennis L Kenyi, Bior K. Bior, Christina Mwangi, Susan Nabadda, Pontiano Kaleebu, Samuel L. Yingst, Prisca Chikwanda, Levi Veri, Raivi Simbi, Heather Alexander

**Affiliations:** US Centers for Disease Control and Prevention (CDC), Atlanta, Georgia, USA (E. Rottinghaus Romano, K. Sleeman, P. Hall-Eidson, C. Zeh, R. Bhairavabhotla, G. Zhang, A. Adhikari, H. Alexander);; Office of the Global AIDS Coordinator, Washington, DC, USA (G. Alemnji);; CDC Center for Global Health, Luanda, Angola (Y. Rebello Cardo);; African Field Epidemiology Network, Luanda (A. Pinheiro);; Instituto Nacional de Luta Contra SIDA, Luanda (B. Pocongo);; CDC Center for Global Health, Yaoundé, Cameroon (L.T. Eno, J.D. Shang, C.B. Ndongmo);; CDC Center for Global Health, Santo Domingo, Dominican Republic (H. Rosario);; Laboratorio Nacional de Salud Pública Dr. Fernando A. Defilló, Santa Domingo (O. Moreno, L.A. De La Cruz De León);; CDC Center for Global Health, Kinshasa, Democratic Republic of the Congo (P. Fonjungo, C. Kabwe);; Institut National de Recherche Biomédicale, Kinshasa (S. Ahuke-Mundeke);; CDC Center for Global Health, Mbabane, Eswatini (D. Gama);; Ministry of Health Mbabane National Reference Laboratory, Mbabane (S. Dlamini, G. Maphalala);; Columbia University Mailman School of Public Health International Center for AIDS Care and Treatment Programs, Mbabane (T. Abreha);; CDC Center for Global Health, Addis Ababa, Ethiopia (A. Purfield, Y.T Gebrehiwot);; Ethiopian Public Health Institute, Addis Ababa (D.M. Desalegn);; CDC Center for Global Health, Nairobi, Kenya (F. Basiye, J. Mwangi);; National Public Health Reference Laboratories, Nairobi (N. Bowen);; CDC Center for Global Health, Maseru, Lesotho (Y. Mengistu, S. Lecher);; CDC Center for Global Health, Lilongwe, Malawi (E. Kampira, M. Kaba);; Ministry of Health Technical Support Services, Lilongwe (J. Bitilinyu-Bangoh);; CDC Center for Global Health, Maputo, Mozambique (G. Masamha);; Ministério da Saúde Instituto Nacional de Saúde, Maputo (S. Omar Viegas);; CDC Center for Global Health, Windhoek, Namibia (R.S. Beard);; Namibia Institute of Pathology Limited, Windhoek (G. van Rooyen, A.N. Shiningavamwe);; CDC Center for Global Health, Abuja, Nigeria (M.I.J. Okoye, N.C. Iriemenam);; Nigeria Centre for Disease Control, Abuja (N. Mba, C. Okoi);; CDC Center for Global Health, Juba, South Sudan (J. Katoro, D.L. Kenyi);; Ministry of Health Public Health Emergency Operations Center, Juba (B.K. Bior);; CDC Center for Global Health, Kampala, Uganda (C. Mwangi);; Ministry of Health National Health Laboratories and Diagnostic Services, Kampala, Uganda (S. Nabadda);; Uganda Virus Research Institute, Entebbe, Uganda (P. Kaleebu);; CDC Center for Global Health, Lusaka, Zambia (S.L. Yingst);; CDC Center for Global Health, Harare, Zimbabwe (P. Chikwanda);; Biomedical Research and Training Institute, Harare (L. Veri);; Zimbabwe Ministry of Health and Child Care, Harare (R. Simbi)

**Keywords:** HIV, tuberculosis, SARS-CoV-2, laboratories, molecular diagnostic techniques COVID-19, coronavirus disease, severe acute respiratory syndrome coronavirus 2, viruses, respiratory infections, zoonoses, vaccine-preventable diseases, PEPFAR

## Abstract

The US President’s Emergency Plan for AIDS Relief (PEPFAR) supports molecular HIV and tuberculosis diagnostic networks and information management systems in low- and middle-income countries. We describe how national programs leveraged these PEPFAR-supported laboratory resources for SARS-CoV-2 testing during the COVID-19 pandemic. We sent a spreadsheet template consisting of 46 indicators for assessing the use of PEPFAR-supported diagnostic networks for COVID-19 pandemic response activities during April 1, 2020, to March 31, 2021, to 27 PEPFAR-supported countries or regions. A total of 109 PEPFAR-supported centralized HIV viral load and early infant diagnosis laboratories and 138 decentralized HIV and TB sites reported performing SARS-CoV-2 testing in 16 countries. Together, these sites contributed to >3.4 million SARS-CoV-2 tests during the 1-year period. Our findings illustrate that PEPFAR-supported diagnostic networks provided a wide range of resources to respond to emergency COVID-19 diagnostic testing in 16 low- and middle-income countries.

Since its inception in 2003, the US President’s Emergency Plan for AIDS Relief (PEPFAR) program has supported >50 countries in their ongoing response to the global HIV and AIDS epidemic, including 22 countries with ongoing HIV and tuberculosis (TB) co-epidemics (*1*). PEPFAR has routinely supported molecular HIV and TB public health laboratory systems and diagnostic networks in low- and middle-income countries (LMICs) to promote patient access to quality clinical testing services and associated care.

SARS-CoV-2, the causative agent of COVID-19, first emerged from China in late 2019 and subsequently spread across the globe. COVID-19 was officially characterized as a pandemic by the World Health Organization on March 11, 2020 (*2*). PEPFAR was quick to respond to this public health emergency and provided the first PEPFAR technical guidance in the context of the COVID-19 pandemic in March 2020 (*3*). That guidance included recommendations on continuity of essential HIV and TB services while ensuring a safe healthcare environment for clients and staff, as well as guidance on the use of PEPFAR-supported resources such as diagnostic networks for the COVID-19 response (*3*).

At the beginning of the epidemic, availability of quality test materials and testing sites was scarce, especially in LMICs (*4*). As SARS-CoV-2 assays became available in LMICs, PEPFAR-supported countries developed and implemented individualized testing strategies that used existing laboratory infrastructure, national laboratory strategic plans, laboratory documentation, standard operating procedures, instrumentation, sample referral networks, supply chain systems, and human resource and technical capacity to perform SARS-CoV-2 testing. These testing strategies were unique to each country and had to balance the SARS-CoV-2 and existing diagnostic testing needs with the availability of reagents and capacity of laboratories to perform the necessary testing within an appropriate timeframe. To achieve the necessary balance, countries used high-throughput centralized laboratories that can test a large number of specimens or lower-throughput decentralized laboratories that are often closer to the point of patient care. We therefore sought to identify and describe the range and quantity of existing centralized and decentralized PEPFAR-supported public health laboratory resources used in response to the COVID-19 pandemic.

## Methods

### Study Design

We designed a retrospective and cross-sectional study by using an information-gathering tool based on Excel (Microsoft, https://www.microsoft.com) to quantify the use of PEPFAR-supported diagnostic networks in LMICs for the COVID-19 response during April 1, 2020–March 31, 2021. We defined a PEPFAR-supported laboratory as a laboratory directly receiving any of the following: infrastructure support or upgrades; molecular testing instrumentation, maintenance, or both; HIV viral load (VL), HIV early infant diagnosis (EID), or TB commodities; human resource or training support; and quality assurance or remote or in-country technical assistance from the PEPFAR program. We identified 3 main use categories: centralized HIV VL and EID instrumentation for SARS-CoV-2 molecular testing; PEPFAR-supported laboratory information systems (LISs) for SARS-CoV-2 laboratory data management; and decentralized HIV VL, HIV EID, and TB instrumentation and resources for SARS-CoV-2 molecular testing on Cepheid GeneXpert instruments (Cepheid, https://www.cepheid.com). We sent the Microsoft Excel tool electronically as an open request to CDC PEPFAR laboratory advisors from 24 countries and 3 regions across the Americas, Africa, and Asia. Data were collected through CDC in-country laboratory advisors during June–August 2021 and verified for completion and quality by CDC headquarters staff in Atlanta, Georgia, USA. This activity was reviewed by CDC and was conducted consistent with applicable federal law and CDC policy. We obtained national SARS-CoV-2 testing volumes from Our World in Data, a publicly available database (*5*).

### Centralized and Decentralized Testing

We defined centralized laboratories as those with high-throughput testing platforms routinely used for HIV VL and EID testing that could also be used for SARS-CoV-2 molecular testing (*6*–*11*). Such platforms included the Abbott m2000 and Alinity M System (Abbott Molecular, https://www.abbott.com), the Roche cobas 6800 and cobas 8800 Systems (Roche Diagnostics, https://diagnostics.roche.com), and the Hologic Panther System (https://www.hologic.com). Decentralized testing sites were defined as those equipped with Cepheid GeneXpert instruments of any modular capacity directly or indirectly supported by the PEPFAR program for TB testing, HIV VL, HIV EID testing, or all of these. We collected country-specific aggregate data on the number of PEPFAR-supported centralized and decentralized laboratories; the number of these laboratories performing SARS-CoV-2 testing; the number of instruments; the volumes of HIV VL and EID, TB, and SARS-CoV-2 testing at centralized and decentralized laboratories; and the use of PEPFAR-supported testing staff, laboratory documentation, training or training materials, commodities and supplies, and LISs or diagnostic connectivity solutions for centralized and decentralized SARS-CoV-2 testing.

### Laboratory Information Systems 

We defined PEPFAR support for a LIS as support for the development, implementation, or maintenance of a LIS. We counted only countries using the adapted PEPFAR-supported LIS for managing SARS-CoV-2 specimens in the laboratory as having implemented the system. We defined the implementation date as the month and year that the first laboratory began recording specimens in the LIS. Countries also reported on the primary format in which the LIS returns results to the clinic and how data from the LIS are shared with the COVID-19 surveillance system.

### Data Analysis

We analyzed and visualized completed tools by using Microsoft Power BI Desktop version 2.96.701.0 (August 2021). Descriptive analyses were conducted by CDC staff at headquarters after verification of data.

## Results

### Overview of PEPFAR Laboratory Support for COVID-19

Sixteen PEPFAR-supported countries responded to the survey, including the Dominican Republic and 15 countries from sub-Saharan Africa. (Table 1). This geographic distribution is fairly representative of the PEPFAR laboratory program, with most support focused in sub-Saharan Africa. All 16 countries reported using the PEPFAR-supported centralized and decentralized laboratories or laboratory resources for SARS-CoV-2 testing, and 11 reported using a PEPFAR-supported HIV VL and EID LIS for SARS-CoV-2 (Kenya used >1 LIS) (Table 1). Of the 11 countries or regions that did not provide data, 4 did not respond to the request, 2 declined to participate because PEPFAR resources were not being used for SARS-CoV-2 testing during the study period, and the remaining 5 could not provide data within the requested timeframe.

**Table 1:** Types of PEPFAR-supported laboratory systems used by 16 countries in their response to the COVID-19 pandemic, April 1, 2020–March 31, 2021*

Country	Centralized resources	Decentralized resources	Laboratory information system
No. (%) countries implementing	16 (100)	16 (100)	11 (73)
Eswatini	✓	✓	✓
Kenya	✓	✓	✓
Lesotho	✓	✓	✓
Malawi	✓	✓	✓
Mozambique	✓	✓	✓
South Sudan	✓	✓	✓
Uganda	✓	✓	✓
Zambia	✓	✓	✓
Nigeria	✓	✓	✓
Namibia	✓	✓	✓
Zimbabwe	✓	✓	✓
Angola	✓	✓	No data
Cameroon	✓	✓	–
Dominican Republic	✓	✓	–
Ethiopia	✓	✓	–
Democratic Republic of the Congo	✓	✓	–

*PEPFAR, US President’s Emergency Plan for AIDS Relief; ✓, use of network component reported; –, network component was not used.

### Centralized Testing

Of the 16 countries that responded, 15 countries reported using PEPFAR centralized VL and EID laboratories for SARS-CoV-2 testing and 1 country (South Sudan) reported no use of those resources (Table 2). Of the 14 countries that reported a date for SARS-CoV-2 test initiation, 8 reported testing for SARS-CoV-2 by April 2020. Five countries (Angola, Malawi, Uganda, Zambia, and Zimbabwe) used 100% of their PEPFAR-supported centralized testing laboratories for SARS-CoV-2 testing (Table 2). Four countries (Democratic Republic of the Congo, Kenya, Cameroon, and Ethiopia) adapted 75%–90% of centralized laboratories for SARS-CoV-2 testing (Table 2). Four countries (Lesotho, Namibia, Nigeria, and Mozambique) used 30%–50% of their PEPFAR-supported centralized laboratories for SARS-CoV-2 testing, and 2 countries (Dominican Republic and Eswatini) used 25% of their centralized laboratories (Table 2). Across the 16 countries, a total of 109 (71%) PEPFAR-supported centralized VL and EID laboratories conducted SARS-CoV-2 testing on 121 centralized VL and EID instruments during the reporting period (Table 2).

**Table 2:** PEPFAR-supported centralized VL and EID laboratories and instruments used for SARS-CoV-2 testing in 16 countries in their response to the COVID-19 pandemic, April 1, 2020–March 31, 2021*

Country	No. PEPFAR laboratories	No. (%) PEPFAR laboratories conducting SARS-CoV-2 testing	No. instruments	No. HIV VL and EID tests conducted in PEPFAR laboratories†	No. SARS-CoV-2 tests conducted in PEPFAR laboratories	No. SARS-CoV-2 tests conducted nationally (*5*)	% SARS-CoV-2 tests performed at PEPFAR laboratories‡
Angola	2	2 (100)	2	NA	No data	NA	NA
Cameroon	13	10 (77)	7	NA	No data	NA	NA
DR	4	1 (25)	1	26,930	588,736	1,176,196§	50
DRC	6	5 (83)	2	176,249	5,565	No data	NA
Eswatini	4	1 (25)	1	NA	No data	NA	NA
Ethiopia	20	15 (75)	15	325,276	630,119	2,355,880¶	27
Kenya	10	8 (80)	25	1,348,294	401,402	571,413#	70
Lesotho	6	3 (50)	0	189,631	47,006	No data	NA
Malawi	11	11 (100)	18	580,578	113,738	56,987¶	200
Mozambique	16	5 (31)	5	1,061,555	378,029	472,224#	80
Namibia	8	4 (50)	3	NA	No data	NA	NA
Nigeria	12	4 (33)	10	1,987,452	208,317	702,055§	30
South Sudan	1	0 (0)	0	NA	0	NA	NA
Uganda	1	1 (100)	11	1,459,010	279,176	851,514§	33
Zambia	24	24 (100)	10	1,025,000	600,000	1,218,207¶	49
Zimbabwe	15	15 (100)	11	650,423	89,504	428,121#	21
Total	153	109 (71)	121	8,830,398	3,341,592	7,832,597	42

*DR, Dominican Republic; DRC, Democratic Republic of Congo; EID, early infant diagnosis; NA, not applicable; PEPFAR, US President’s Emergency Plan for AIDS Relief; VL, viral load. †Number of HIV VL and EID and national SARS-CoV-2 tests are only shown for those countries reporting SARS-CoV-2 testing volumes in PEPFAR-supported laboratories. For countries not reporting SARS-CoV-2 testing volumes in PEPFAR-supported laboratories, HIV VL and EID and national SARS-CoV-2 test numbers are listed as NA. ‡ Percentage of SARS-CoV-2 tests performed at PEPFAR-supported laboratories was only calculated for countries with data available for both PEPFAR and national SARS-CoV-2 testing numbers. For countries without both PEPFAR and national SARS-CoV-2 testing numbers available, % of SARS-CoV-2 tests performed at PEPFAR laboratories is listed as NA. §National SARS-CoV-2 test numbers represent the number of PCR tests. ¶Test type for national SARS-CoV-2 test numbers was uncited or listed as unclear. #National SARS-CoV-2 test numbers represent the number of PCR and antigen tests.

Of the 15 countries reporting SARS-CoV-2 testing at PEPFAR-supported centralized VL and EID laboratories, 11 reported SARS-CoV-2 testing volumes. In these 11 countries, a total of 3,341,592 SARS-CoV-2 tests were performed in PEPFAR-supported centralized VL and EID laboratories during the 12-month reporting period and accounted for 42% of the national testing volumes in these countries according to a publicly available database (*5*) (Table 2). Three countries (Ethiopia, Zambia, and Dominican Republic) performed >500,000 SARS-CoV-2 tests using PEPFAR-supported laboratories during the study period, contributing to 27% (Ethiopia), 49% (Zambia), and 50% (Dominican Republic) of the national testing volumes (Table 2). These countries also had the highest proportion of SARS-CoV-2 tests performed in PEPFAR-supported centralized laboratories compared with HIV VL and EID testing ranging from 96% in the Dominican Republic to 37% in Zambia (Table 2). Four countries (Kenya, Mozambique, Uganda, and Nigeria) performed ≈208,000–402,000 SARS-CoV-2 tests during the reporting period, contributing to 70% (Kenya), 80% (Mozambique), 33% (Uganda), and 30% (Nigeria) of the national SARS-CoV-2 testing volume (Table 2). These countries also performed >1 million HIV VL and EID tests each (Table 2). Angola, Cameroon, Eswatini, Namibia, and South Sudan did not report SARS-CoV-2 test volumes.

Thus far, we have described the contribution of physical laboratory space and instrumentation to SARS-CoV-2 testing in PEPFAR-supported centralized laboratories. We assessed additional categories of centralized laboratory support provided by PEPFAR and whether they were used for SARS-CoV-2 testing (Table 3). Of the 16 countries, 14 reported using laboratory documentation for SARS-CoV-2 testing, 13 reported using testing staff, 12 reported using the specimen referral network, and 10 countries each reported using PEPFAR-supported laboratory training materials (Table 3). Three countries that reported no use of PEPFAR testing staff to conduct SARS-CoV-2 testing indicated that trained ministry of health staff conducted the testing in these laboratories. Although it was not requested, a few countries provided additional information on PEPFAR resources contributing to SARS-CoV-2 external quality-assurance programs.

**Table 3:** Use of PEPFAR-supported centralized VL and EID diagnostic networks for SARS-CoV-2 testing in 16 countries in their response to the COVID-19 pandemic, April 1, 2020–March 31, 2021*

Country	Testing staff	Laboratory documentation	Training materials	Specimen referral networks
No. (%) countries implementing	13 (81)	14 (93)	10 (67)	12 (75)
Kenya	✓	✓	✓	✓
Malawi	✓	✓	✓	✓
Mozambique	✓	✓	✓	✓
Namibia	✓	✓	✓	✓
Nigeria	✓	✓	✓	✓
South Sudan†	✓	✓	✓	✓
Zimbabwe	✓	✓	✓	✓
Cameroon	✓	No data	No data	✓
Democratic Republic of Congo	✓	✓	✓	–
Uganda	✓	✓	–	✓
Lesotho	✓	✓	–	✓
Zambia	✓	✓	–	✓
Angola	–	✓	✓	–
Dominican Republic	–	✓	✓	–
Eswatini	–	✓	–	✓
Ethiopia	✓	–	–	–

*PEPFAR, US President’s Emergency Plan for AIDS Relief; ✓, use of network component reported; –, network component was not used. †Did not report utilizing PEPFAR-supported centralized viral load and early infant diagnosis laboratories for SARS-CoV-2 testing.

### Decentralized Testing

As with centralized HIV molecular testing instrumentation, modular GeneXpert near-point-of-care systems are designed for multi-disease testing. By March 31, 2021, five countries had not introduced the Xpert Xpress SARS-CoV-2 assay into their networks, in part because of disruptions in the availability of GeneXpert testing services (Angola) or national implementation plans prioritizing high-volume centralized testing strategies or test implementation at sites outside the PEPFAR-supported network (Ethiopia, Kenya, Uganda, and Cameroon) (Table 4). The remaining 11 countries reported integration of SARS-CoV-2 into GeneXpert-based TB or TB and HIV services across a total of 138 (7.1%) PEPFAR-supported decentralized molecular sites (Table 4). Of note, decentralized SARS-CoV-2 testing was not reported in any country until June 2020 likely because of the reasons stated previously.

**Table 4:** PEPFAR-supported decentralized laboratories and instruments used for SARS-CoV-2 testing in 16 countries in their response to the COVID-19 pandemic, April 1, 2020–March 31, 2021*

Country	No. PEPFAR sites	No. (%) PEPFAR sites conducting SARS-CoV-2 testing	No. TB tests conducted in PEPFAR sites†	No. HIV VL and EID tests conducted in PEPFAR sites†	No. SARS-CoV-2 tests conducted in PEPFAR sites	No. SARS-CoV-2 tests conducted nationally	% SARS-CoV-2 tests performed at PEPFAR sites‡
Angola	4	0 (0)	NA	NA	No data	NA	NA
Cameroon	13	0 (0)	NA	NA	No data	NA	NA
DR	11	7 (64)	18,519	3,133	1,240	1,176,196§	0.1
DRC	18	2 (11)	NA	NA	No data	No data	NA
Eswatini	32	1 (3)	18,243	1,196	873	No data	NA
Ethiopia	280	0 (0)	NA	NA	No data	NA	NA
Kenya	158	0 (0)	NA	NA	No data	NA	NA
Lesotho	33	3 (9)	19,596	15,046	21,946	No data	NA
Malawi	89	35 (39)	33,450	43,602	10,482	56,987¶	18.4
Mozambique	161	6 (4)	159,685	0	10,332	472,224#	2.2
Namibia	45	4 (9)	NA	NA	No data	NA	NA
Nigeria	400	27 (7)	56,183	0	39,902	702,055§	5.7
South Sudan	17	17 (100)	4,024**	1,081**	2,931**	41,171¶	7.1
Uganda	250	0 (0)	NA	NA	No data	NA	NA
Zambia	300	3 (1)	150,000	6,000	27,000	1,218,207¶	2.2
Zimbabwe	122	33 (27)	8,326	1,247	9,976	428,121#	2.3
Total	1,933	138 (7.1)	468,026	71,305	124,682	4,094,961	2.5

*DR, Dominican Republic; DRC, Democratic Republic of Congo; EID, early infant diagnosis; NA, not applicable; PEPFAR, US President’s Emergency Plan for AIDS Relief; VL, viral load. †Number of HIV VL and EID and national SARS-CoV-2 tests are only shown for those countries reporting SARS-CoV-2 testing volumes in PEPFAR-supported laboratories. For countries not reporting SARS-CoV-2 testing volumes in PEPFAR-supported laboratories, HIV VL and EID and national SARS-CoV-2 test numbers are listed as NA. ‡Percentage of SARS-CoV-2 tests performed at PEPFAR-supported laboratories was only calculated for countries with data available for both PEPFAR and national SARS-CoV-2 testing numbers. For countries without both PEPFAR and national SARS-CoV-2 testing numbers available, % of SARS-CoV-2 tests performed at PEPFAR laboratories is listed as NA. §National SARS-CoV-2 test numbers represent the number of PCR tests. ¶Test type for national SARS-CoV-2 test numbers was uncited or listed as unclear. #National SARS-CoV-2 test numbers represent the number of PCR and antigen tests. **Testing numbers reported in South Sudan PEPFAR laboratories represent the period October 2020–March 2021.

Although the number of PEPFAR-supported GeneXpert laboratories varied by country, South Sudan (17/17 [100%]), Dominican Republic (7/11 [64%]), Malawi (35/89 [39%]), Zimbabwe (33/122 [27%]), and the Democratic Republic of the Congo (2/18 [11%]) reported the highest proportion of PEPFAR-supported decentralized instruments used for SARS-CoV-2 testing (Table 4). The remaining countries used <10% of their PEPFAR-supported decentralized networks for SARS-CoV-2 (Table 4). As expected, the proportion of GeneXpert network use generally correlated with network size; the highest proportion of instruments used was reported by countries with <125 instruments, whereas lower proportions were reported by countries supporting larger networks, such as Nigeria, Zambia, Ethiopia, and Uganda (250–400 instruments) (Table 4).

Of the 11 countries that introduced SARS-CoV-2 testing at PEPFAR-supported GeneXpert sites, 9 reported testing volumes for TB and SARS-CoV-2, of which 7 also indicated the provision of GeneXpert-based HIV VL or EID testing services and reported combined HIV-specific testing volumes (Table 4). The highest SARS-CoV-2 testing volumes were reported from Nigeria (39,902 tests), Zambia (27,000 tests), and Lesotho (21,946 tests), followed by Malawi (10,482 tests), Mozambique (10,332 tests), and Zimbabwe (9,976 tests), whereas the lowest testing volumes were reported from Dominican Republic (1,240) and Eswatini (873) (Table 4). Similarly, South Sudan reported a low testing volume for the portion of the reporting period for which testing data were available (2,931 tests during October 2020–March 2021) (Table 4). Because of lower instrument throughput, PEPFAR-supported decentralized sites contributed to a small percentage of the national SARS-CoV-2 testing volumes (2.5%) in the countries with data available (Table 4). Of note, most (6/8 [75%]) of reporting countries completed more TB tests than HIV or SARS-CoV-2 tests during the pandemic period, ranging from 43% to 94% of testing by country conducted during the reporting period (Table 4). Only Malawi and Lesotho indicated higher volumes of HIV tests (50% in Malawi) and SARS-CoV-2 tests (39% in Lesotho) than either other disease (Table 4), which is in agreement with published reports of reduced TB service use and case notifications in these countries during a period of HIV or SARS-CoV-2 Xpert test scale-up (*12*,*13*). Overall, PEPFAR supported the completion of >664,000 TB, HIV, and SARS-CoV-2 Xpert tests across the 9 reporting countries during the study period.

In addition to the use of GeneXpert instruments and Xpert cartridges at PEPFAR-supported testing sites, 12 of 16 reporting countries reported additional use of other components of the PEPFAR-supported diagnostic network for implementation of the Xpert Xpress SARS-CoV-2 molecular test (Table 5). Support for testing staff to conduct SARS-CoV-2 tests was reported by all 11 countries, followed closely by the use of laboratory documentation to record Xpert Xpress SARS-CoV-2 testing data and trainings or training materials for new or existing site staff (10/12 [83%]) (Table 5). Commodities required for safe and accurate Xpert Xpress SARS-CoV-2 testing were also provided in 8 (67%) countries and included, but were not limited to, personal protective equipment, waste management materials, testing consumables and supplies, and Xpert Check calibration cartridges (Table 5). In addition, 7 countries reported using the PEPFAR-supported diagnostic connectivity solutions to track or report Xpert Xpress SARS-CoV-2 test results to healthcare providers or disease surveillance programs (Table 5). Of note, nearly all the countries that implemented SARS-CoV-2 GeneXpert testing at PEPFAR-supported sites used >4 of the 5 network support components; testing in Eswatini, Lesotho, and Zimbabwe were supported with all listed components by the end of the reporting period (Table 5). Uganda did not report use of PEPFAR-supported GeneXpert sites for SARS-CoV-2 testing but did report use of PEPFAR-supported connectivity solutions (Table 5).

**Table 5:** Use of PEPFAR-supported decentralized diagnostic networks for SARS-CoV-2 testing in 12 countries in their response to the COVID-19 pandemic, April 1, 2020–March 31, 2021*

Country	Testing staff	Laboratory documentation	Training materials	Commodities	Connectivity
No. (%) countries implementing	11 (92)	10 (83)	10 (83)	8 (67)	7 (58)
Eswatini	✓	✓	✓	✓	✓
Lesotho	✓	✓	✓	✓	✓
Zimbabwe	✓	✓	✓	✓	✓
Democratic Republic of the Congo	✓	✓	✓	✓	–
Dominican Republic	✓	✓	✓	✓	–
Nigeria	✓	✓	✓	✓	–
South Sudan	✓	✓	✓	✓	–
Malawi	✓	✓	✓	–	✓
Zambia	✓	✓	–	✓	✓
Namibia	✓	✓	✓	–	–
Mozambique	✓	–	✓	–	✓
Uganda†	–	–	–	–	✓

*PEPFAR, US President’s Emergency Plan for AIDS Relief; ✓, use of network component reported; –, network component was not used. †Uganda did not report using PEPFAR-supported decentralized laboratories for SARS-CoV-2 testing.

### LISs

LISs help to manage specimens and workflows within the laboratory and are critical for ensuring efficient laboratory testing and reporting in high-throughput laboratories. As stated, of the 16 countries reporting data, 11 reported having adapted and implemented the existing PEPFAR-supported LIS (or LISs, as in Kenya) for managing SARS-CoV-2 testing (Table 1) in 121 centralized and decentralized laboratories (Table 6). Reasons for countries reporting not adapting a PEPFAR-supported LIS included implementation of the PEPFAR-supported LIS after the reporting period and use of a non–PEPFAR-supported LIS. The time to adapt the LIS for SARS-CoV-2 testing ranged from March to December 2020; nine of the 11 countries reported the system having been implemented by the end of June 2020 (Table 6). We categorized the type of LISs that were adapted and found that 5 countries (Namibia, Eswatini, Zambia, Lesotho, and Mozambique) adapted a commercial LIS, 2 countries (Uganda and Nigeria) adapted a custom-built LIS, 1 country (Kenya) adapted a mix of commercial and custom-built LISs, and 3 countries (Malawi, Zimbabwe, and South Sudan) adapted open-source LIS solutions (Table 6).

**Table 6:** Summary of PEPFAR-supported LISs adapted for SARS-CoV-2 testing in 11 countries in their response to the COVID-19 pandemic, April 1, 2020–March 31, 2021*

Country	No. PEPFAR-supported laboratories with a SARS-CoV-2 LIS†	LIS category	Month and year the SARS-CoV-2 LIS was implemented in first laboratory
Namibia	39	Commercial	Mar 2020
Mozambique	16	Commercial	Mar 2020
Eswatini	2	Commercial	Apr 2020
Zambia	24	Commercial	Apr 2020
Nigeria	4	Custom-built	Apr 2020
Uganda	3	Custom-built	May 2020
Malawi	5	Open-source	Jun 2020
Zimbabwe	15	Open-source	Jun 2020
Kenya	8	Commercial and custom-built	Jun 2020
Lesotho	3	Commercial	Dec 2020
South Sudan	2	Open-source	Dec 2020
Total	121		

*LIS, laboratory information system; PEPFAR, US President’s Emergency Plan for AIDS Relief. †Number of PEPFAR-supported laboratories with a SARS-CoV-2 LIS includes centralized and decentralized laboratories.

One benefit of an LIS is the ability to return results to the clinic through a paperless route and thus decrease the turnaround time for this segment of laboratory testing. Nine (82%) of the 11 countries using a PEPFAR-supported LIS for SARS-CoV-2 testing returned results through electronic means (8 countries) or through SMS (1 country), whereas 2 (18%) countries reported returning results through a paper system. We should note that these means of result return represent the primary format and that several countries reported using various methods on the basis of the capacity of the health facilities receiving the results.

In addition to returning results efficiently, LISs are also used for surveillance purposes by providing the number and (potentially) demographic information of patients or samples tested and the results of those tests. All 11 countries reported that the LIS contributed to COVID-19 surveillance; 4 (36%) 11 of countries reporting exporting data from the LIS directly (1 country) or indirectly (3 countries) to an electronic medical record or surveillance system, and 6/11 (55%) described exporting data from the LIS in bulk for surveillance purposes. The remaining country reported manual entry of results from an LIS to the surveillance system.

## Discussion

Worldwide, more COVID-19 cases were documented in the first 5 months of 2021 than in all of 2020 (*5*). Many PEPFAR-supported countries experienced multiple waves of infections. Although challenges facing LMICs in battling the ongoing COVID-19 pandemic are numerous (*14*), existing PEPFAR-supported diagnostic networks and ongoing laboratory strengthening activities enabled several countries to effectively respond to emergency SARS-CoV-2 testing demands in a timely manner.

SARS-CoV-2 testing volumes on PEPFAR-supported centralized and decentralized molecular instruments were dependent on each country’s individualized COVID-19 testing strategy, which considered many factors, including instrument capacity, availability of SARS-CoV-2 molecular test kits, reagents and consumables, availability of trained staff, and total testing need for all diseases on each instrument or in each laboratory. Furthermore, centralized and decentralized testing offer unique benefits; centralized testing offers higher testing volumes, and decentralized testing offers increased testing access nearer to the patient. For those reasons, laboratory use and testing volumes between countries or laboratory types cannot be meaningfully compared; however, our findings demonstrate that existing PEPFAR-supported centralized and decentralized diagnostic networks contributed to SARS-CoV-2 testing in all countries reporting data and to 43% of national testing volumes reported in a publicly available database (Tables 2, 4) (*5*). This contribution was potentially even higher given that PEPFAR testing data were limited to molecular tests and the testing data for several countries in the database included antigen testing or did not specify the type of test reported (*5*). We should note that SARS-CoV-2 testing in PEPFAR-supported countries and laboratories was probably limited by a global shortage of molecular reagents and test kits that disproportionately affected automated molecular platforms and LMICs (*4*,*15*). Furthermore, PEPFAR only supports closed platforms for molecular testing, and our study therefore did not investigate the use of open platforms for SARS-CoV-2 testing, which were commonly used across LMICs, particularly early in the pandemic. Nevertheless, the PEPFAR-supported contributions to national SARS-CoV-2 testing volumes are substantial and illustrate that PEPFAR-supported laboratory strengthening efforts in LMIC are not only beneficial for HIV- and TB-related programs and services but can have a broader public health benefit.

SARS-CoV-2 testing was performed at PEPFAR-supported centralized and decentralized molecular laboratories in addition to routine HIV VL, HIV EID, and TB testing. For most countries, apart from Ethiopia and the Dominican Republic, SARS-CoV-2 testing accounted for <50% of total centralized and decentralized SARS-CoV-2, HIV VL and EID, and TB testing volumes (Tables 2, 4). Diagnostic networks in LMICs have historically been implemented in a siloed, program-specific manner (*16*), resulting in parallel networks operating or managed by different entities. With the availability of molecular platforms, which can test for multiple diseases, and a need for more sustainable and efficient networks, countries are exploring how to integrate these parallel networks and use instruments to test for several diseases. Data for HIV and TB testing trends before the COVID-19 pandemic were not collected in this study and thus the effect of integration of SARS-CoV-2 on existing test cannot be directly assessed; however, the use of existing laboratories, instrumentation, and sample transport networks within these PEPFAR-supported countries for SARS-CoV-2 testing demonstrates the feasibility of diagnostic network integration. Although diseases and testing needs will differ by country, the process of assessing the existing network infrastructure and capacity and determining how to meet the cumulative testing demand is the same across all countries. Lessons learned from cross-disease resource sharing between TB, HIV, and COVID-19 in these countries and others can guide future models for integrated, patient-centered service delivery.

The variety of categories and types of LISs adapted for SARS-CoV-2 testing in PEPFAR-supported countries illustrate the diversity of the PEPFAR LIS portfolio and the versatility of information system maintenance and support. The diversity of the systems in place is indicative of country-led efforts in LIS selection and implementation. Each of the categories of LISs (commercial, custom built, or open-source) require a different level of upfront and recurring costs to implement and maintain, yet each category was successfully adapted and implemented for SARS-CoV-2 testing in PEPFAR-supported countries (Table 6). These data demonstrate that the countries had, or quickly acquired, the necessary technical and financial support to update their LISs to respond to a global pandemic.

The first limitation of our study is that our analysis is limited to the countries that reported data and thus cannot be extrapolated to the entire PEPFAR program, given that the decision to participate or not provide complete data could have been biased by the level of PEPFAR resources used for SARS-CoV-2. In addition, the reported scope of laboratory resources used by these 16 countries is limited to molecular diagnostic networks and only the resources supported by PEPFAR. Although PEPFAR-supported diagnostic networks are extensive, they are not nationally representative and do not include other disease program laboratory services that were similarly adapted for SARS-CoV-2 molecular testing during the initial year of the COVID-19 pandemic. Furthermore, the level of PEPFAR support for each of the countries varies, and thus the countries cannot be compared to each other. Our analysis might also be limited by the quality of the data reported. Although data were reported in line with the indicators to the best of the individual or country team’s knowledge, reporting errors may have occurred. Nonetheless, our data demonstrate the resiliency of laboratory systems strengthened through PEPFAR and how quickly these systems were able to adapt to accommodate testing for SARS-CoV-2.
